# Eggs and Poultry Purchase, Storage, and Preparation Practices of Consumers in Selected Asian Countries

**DOI:** 10.3390/foods3010110

**Published:** 2014-01-16

**Authors:** Kadri Koppel, Suntaree Suwonsichon, Uma Chitra, Jeehyun Lee, Edgar Chambers

**Affiliations:** 1 The Sensory Analysis Center, Kansas State University, 1310 Research Park Drive, Manhattan, KS 66502, USA; E-Mail: eciv@ksu.edu; 2 Kasetsart University Sensory and Consumer Research Center, Department of Product Development, Kasetsart University, 50 Paholyothin Road, Jatujak, Bangkok 10900, Thailand; E-Mail: fagisrsu@ku.ac.th; 3 Department of Nutrition and Dietetics, Kasturba Gandhi College for Women, Secunderabad 500026, India; E-Mail: umachitra7@gmail.com; 4 Department of Food Science and Nutrition, College of Human Ecology, Pusan National University, 30 Jangjeon-Dong, Geumjeoung-Ku, Busan 609 735, Korea; E-Mail: jeehyunlee@pusan.ac.kr

**Keywords:** food safety, consumer, eggs, poultry

## Abstract

The objective of this study was to begin characterizing purchase, storage, handling, and preparation of poultry products and eggs by selected consumers in three Asian countries: India, Korea, and Thailand. Approximately 100 consumers in each location were recruited to participate in this study. The consumers were surveyed about eggs and poultry purchase behavior characteristics, such as temperatures and locations, storage behavior, such as storage locations in the refrigerator or freezer, preparation behavior, such as washing eggs and poultry before cooking, and handling behavior, such as using cutting boards during cooking. The results indicated differences in purchase and storage practices of raw eggs. Most Korean consumers purchased refrigerated eggs and stored the eggs in the refrigerator, while Indian and Thai consumers bought eggs that were stored at room temperature, but would refrigerate the eggs at home. Approximately half of the consumers in each country froze raw meat, poultry, or seafood. Food preparation practices showed potential for cross-contamination during cooking, such as using the same cutting board for different kinds of foods or not washing hands with soap and water. The results presented in this pilot study may lead to development of educational messages and raising consumer awareness of food safety practices in Asian countries.

## 1. Introduction

One key aspect of food security is food safety; sufficient and available food quantities alone do not guarantee a healthy and nourished population. Foodborne diseases are widespread in the world and consumer education on food safety topics is of high importance [1]. World Health Organization (WHO) has introduced the Five Keys Program [2] that stresses importance of cleanliness, avoiding cross-contamination, cooking and storing at proper temperatures, and using safe water to avoid foodborne diseases. Some of the key food ingredients that are related to salmonellosis outbreaks, if not handled correctly, are eggs and poultry. The main determinants of contamination while handling raw meat and poultry are related to washing practice of contaminated hands, cutting boards, and knives during and after cooking [3]. Egg processing in Asia is the largest in the world, however, in India, egg products consumption is low and production has been reduced. In Korea and Thailand, egg products are being manufactured and quantities are predicted to increase [4]. Furthermore, poultry production and consumption in Asia has been increasing in recent years [5,6]. For example, in India alone, chicken consumption has increased from 1.8 to 2.2 kg/person/year. This seemingly low increase in average consumption becomes critical considering that about 1.2 trillion people live in India.

Consumer behavior regarding eggs and poultry usage has been studied in several countries. For example consumers in Belgium [7], New Zealand [8], the U.S. [9,10], China [11], Brazil [12], Turkey [13], and Greece [14] have been studied regarding their food safety practices while handling eggs and poultry. Food safety concerns in India have been studied with a focus on street food safety and quality [15] and consumer behavior [16,17]. Similarly, in Korea, food safety knowledge, attitudes, or practices were studied among at risk population, such as middle school students [18], the elderly [19,20], and people who prepare food for others, such as kitchen employees at school [21], senior welfare center employees [22], house wives [23], and culinary and hospitality major college students [24,25]. Few studies have done so in Thailand [26]. No studies were found that would compare food safety practices across several Asian countries. 

Some studies have looked at food safety issues around street foods that are common in Asian countries. For example, Sudershan *et al.* [15] studied street foods prepared with poultry in the Hyderabad region of India. They found that several of the foods were contaminated with *Staphylococcus aureus* or/and *Bacillus cereus*. Furthermore, local people did not consider street foods safe and thought that foods prepared at home were more hygienic [17]. Those authors reported that safety of home-prepared meals is ensured by cleanliness of the cook, surroundings, and the food. However in Italy, researchers surveyed food safety practices among consumers to develop food safety education program, based on the belief that the home was a source of foodborne disease [27]. According to Henley *et al.* [9], there may be some culturally unique behaviors related to food handling and preparation among Asian cultures. Examples of those include washing raw poultry with hot water or with acidic solutions and purchasing live poultry. A study comparing food safety issues of consumers from Seoul and Shanghai concluded that culture has an influence on perception and behavior [28]. The benefits of a study that compares consumers from several Asian countries include increased knowledge regarding possible culturally specific behaviors, cross-contamination, spoilage, and resulting food-borne illnesses and a uniform approach to consumers that allows data comparison. The objective of this pilot scale study was to begin to characterize consumers’ purchase, storage, handling, and preparation of poultry products and eggs in three different Asian countries: India, Korea, and Thailand.

## 2. Experimental Section

### 2.1. Questionnaire and Data Collection

The questionnaire was based on questionnaires used in previous research [29], was developed in English, and pretested on food safety experts to ensure collection of relevant information and to ensure uniform understanding of the questions. The questionnaire also was pretested in the U.S. with a group of consumers before translation and with local staff, “in country”, after translation. The questionnaire was translated into Korean, and Thai, and then back-translated into English by native speakers to ensure the questionnaire was comparable across countries. In India, the questionnaire was given in English, as all consumers were fluent in the English language. Data was collected in the beginning of 2013 in India and Thailand, and in the middle of 2013 in Korea. The information was gathered via an online survey (Korea) or at a central location where the consumers filled in paper questionnaires (India and Thailand). 

The questionnaire collected information on a wide range of home storage and handling practices. Only information regarding 11 multiple-choice questions regarding consumers’ eggs and poultry related purchase, storage, preparation, and refrigeration practices are presented. 

Demographic information regarding consumer gender, age (<35; ≥35), household income (equivalent as determined by the country to “Low”: <$25,000; “Medium”: $25,000–$50,000; “High”: >$50,000), and education level (less than college; some college courses or higher) was collected in the end of the questionnaire. Demographic information regarding the consumers who participated in the study is shown in Table 1.

### 2.2. Consumers

Consumers were tested in Bangkok, Thailand; Hyderabad, India; and Busan and Suncheon, Korea; countries where established food and nutrition researchers were available and willing to conduct the test. In addition, consumers in these countries present a broad diversity of Asian consumers. Consumers were selected from those who were already in databases maintained by researchers, from advertisements in newspapers and newsletters, and word-of-mouth referrals and, thus, are a convenience sample rather than a nationally representative sample. The number and location of the consumers cannot represent the entire population of a country, but serves as a “first look” of practices in those countries. 

All participating consumers were pre-screened according to the following conditions: age >18; the consumer was the primary food shopper in the household or shared food shopping responsibility with someone else; the consumer was the food preparer and knew about food storage in the household or was one of several people who cooked and knew about food storage in household; the consumer had a refrigerator in their home. A total of 115 consumers participated in India, 100 in Thailand, and 101 in Sunchoen, Korea. Only consumers who were food shoppers and food preparers were included in this survey, and this limits the consumers available for sampling. In India, all consumers, and in Korea and Thailand most surveyed consumers were female (Table 1) because food preparation in those countries typically is a task to be conducted by females in the household. In addition, because refrigeration is a key aspect of suggested egg and poultry storage, only those consumers with access to a refrigerator were selected. In India, in particular, this decreased potential participation, but consumers without a refrigerator, by default, could not store poultry or eggs as recommended.

**Table 1:** Demographic characteristics of the surveyed consumers.

Demographic segmentation, %	India (*n* = 115)	Korea (*n* = 101)	Thailand (*n* = 100)
*Gender*			
Male		15.0	27.0
Female	100.0	86.0	73.0
*Age*			
<35	28.7	35.0	13.0
>35	71.3	66.0	87.0
*Education*			
Less than college	21.7	39.0	23.0
Some college courses or more	78.3	62.0	77.0
*Income*			
Low	46.0	24.0	3.0
Medium	27.0	33.0	36.0
High	27.0	44.0	61.0

### 2.3. Data Analysis

Chi-square tests were performed for the relationships between various sociodemographic variables. The analysis was conducted using Excel function CHITEST (Microsoft Excel 2010, Microsoft, Redmond, WA, USA).

## 3. Results and Discussion

### 3.1. Purchase and Storage Practices

Most surveyed consumers in India, Thailand, and Korea purchased eggs from the store or supermarket (Table 2). Less than 10% of respondents in each country purchased eggs directly from farmers or raised their own chicken. In addition 32% of Thai consumers purchased eggs from the market. In India, respondents who were older than 35 years were significantly more likely to buy eggs from the market than younger consumers (*p* = 0.03). This might imply a heightened risk of pathogens on eggshells as a washing step often is not conducted for eggs sold at markets. Total viable counts of microorganisms, from eggshell, have been found to be significantly higher on unwashed eggshell compared to washed eggshell [30].

**Table 2:** Consumers’ egg purchase location. Where do you usually buy eggs?

Demographic segmentation, %	India (*n* = 115)			Korea (*n* = 101)			Thailand (*n* = 100)		
	Farmer/raise chicken	Store	Market	Farmer/raise chicken	Store	Market	Farmer/raise chicken	Store	Market
Total	2.6	75.7	21.7	6.9	74.2	8.9	5.0	63.0	32.0
*Gender*									
Male					13.9	1.0	1.0	18.0	8.0
Female	2.6	75.7	21.7	6.9	70.3	7.9	4.0	45.0	24.0
*Age*									
<35	**0.9**	**21.7**	**6.1**	3.0	26.7	5.0		9.0	4.0
>35	**1.7**	**53.9**	**15.7**	4.0	57.4	4.0	5.0	54.0	28.0
*Education*									
Less than college		14.8	7.0	**4.0**	**27.7**	**6.9**	3.0	15.0	2.0
Some college courses or more	2.6	60.9	14.8	**3.0**	**56.4**	**2.0**	2.0	48.0	27.0
*Income*									
Low	0.9	33.9	11.3	**2.0**	**14.9**	**6.9**		2.0	1.0
Medium	1.7	19.1	6.1	**1.0**	**31.7**	**0.0**	5.0	22.0	9.0
High		22.6	4.3	**4.0**	**37.6**	**2.0**		39.0	22.0

Note: values shown in bold are statistically significantly different among those sociodemographic portions according to chi-square test for (*p* < 0.05).

At the time of purchase the eggs were usually refrigerated in Korea, most likely because the Korean Ministry of Food and Drug Safety regulates the distribution of eggs and requires them to be at 0–15 °C [31]. However, in Thailand and India eggs were at room temperature when purchased (Table 3). In addition 16% of consumers in India, 10% of consumers in Korea, and 16% of consumers in Thailand stored raw eggs at room temperature (Table 4). These practices could be related to inclusion of lacto-ovo vegetarians in the study, whose households have only small refrigerators for the most perishable products, but also could subject some Indian, Thai, and Korean consumers to a higher risk for foodborne diseases. Salmonellosis is a leading cause of foodborne diseases throughout the world [32]. A previous study has shown that bacteria causing salmonellosis can multiply within eggsand reach high concentrations when stored at room temperature [33]; this could be exacerbated in warmer climates. In addition, it was reported that the Haugh unit, which indicates freshness of egg albumen, was negatively influenced by warmer temperature [30]. According to modeling studies [34] the risk of foodborne illness is reduced if the shelf life is less than seven days. 

**Table 3:** Percentage of eggs refrigerated at time of purchase. When you usually buy eggs are they refrigerated or at room temperature?

Demographic segmentation, %	India (*n* = 115)		Korea (*n* = 101)		Thailand (*n* = 100)	
	Refrigerated	Room temperature	Refrigerated	Room temperature	Refrigerated	Room temperature
Total		100.0	78.2	22.8	36.0	64.0
*Gender*						
Male			12.9	3.0	8.0	19.0
Female		100.0	65.3	19.8	28.0	45.0
*Age*						
<35		28.7	28.7	5.9	**9.0**	**4.0**
>35		71.3	48.5	16.8	**27.0**	**60.0**
*Education*						
Less than college		21.7	31.7	6.9	6.0	17.0
Some college courses or more		78.3	45.5	15.8	30.0	47.0
*Income*						
Low		46.1	17.8	5.9	1.0	2.0
Medium		27.0	26.7	5.9	14.0	22.0
High		26.9	32.7	10.9	21.0	40.0

Note: values shown in bold are statistically significantly different among those sociodemographic portions according to chi-square test for (*p* < 0.05).

**Table 4:** Consumers’ storage behavior of raw eggs in the shell. Where would you store the food items?

Demographic segmentation, %	India (*n* = 115)			Korea (*n* = 94) *		Thailand (*n* = 100) *	
	Refrigerator	Room temperature	Do not know	Refrigerator	Room temperature	Refrigerator	Room temperature
Total	81.7	16.5	1.7	89.4	10.6	74.0	16.0
*Gender*							
Male				16.0		22.0	5.0
Female	81.7	16.5	1.7	73.4	10.6	62.0	11.0
*Age*							
<35	25.2	3.5		**36.2**	**1.1**	12.0	1.0
>35	56.5	13.0	1.7	**53.2**	**9.6**	72.0	15.0
*Education*							
Less than college	16.5	4.3	0.9	**27.7**	**7.4**	18.0	5.0
Some college courses or more	65.2	12.2	0.9	**61.7**	**3.2**	66.0	11.0
*Income*							
Low	42.6	7.8	1.7	17.0	3.2	3.0	
Medium	21.7	5.2		29.8	4.3	30.0	6.0
High	23.5	3.5		42.6	3.2	51.0	10.0

Notes: values shown in bold are statistically significantly different among those sociodemographic portions according to chi-square test for (*p* < 0.05). * No consumers in Korea and Thailand answered “Do not know” option.

When it comes to storing cooked eggs in the shell, behavior became more diverse. For example 37% of surveyed Indian consumers stored cooked eggs in the shell at room temperature and 25% of the Indian consumers didn’t know how to store cooked eggs in the shell (Table 5). Furthermore, 34% of surveyed Korean and 42% of Thai consumers stored cooked eggs in the shell at room temperature. Korean consumers older than 35 were significantly more likely to store cooked eggs in the refrigerator than younger consumers, more of whom did so at room temperature (*p* = 0.004). 

**Table 5:** Consumers’ storage behavior of cooked eggs in the shell. Where would you store the food items?

Demographic segmentation, %	India (*n* = 115)			Korea (*n* = 91)			Thailand (*n* = 99)		
	Refrigerator	Room temperature	Do not know	Refrigerator	Room temperature	Do not know	Refrigerator	Room temperature	Do not know
Total	37.4	37.4	25.2	64.8	34.1	1.1	55.6	42.4	2.0
*Gender*									
Male				8.8	7.7		14.1	12.1	
Female	37.4	37.4	25.2	56.0	26.4	1.1	41.5	30.3	2.0
*Age*									
<35	10.4	11.3	7.0	**17.6**	**20.9**		8.1	4.0	
>35	27.0	26.1	18.3	**46.2**	**13.2**	**1.1**	47.5	38.4	2.0
*Education*									
Less than college	**4.3**	**13.0**	**4.3**	22.0	11.0	1.1	11.1	10.1	1.0
Some college courses or more	**33.0**	**24.3**	**20.9**	42.9	23.1		44.5	32.3	1.0
*Income*									
Low	18.3	20.0	7.8	12.1	5.5	1.1	1.0	2.0	
Medium	9.6	9.6	7.8	24.2	9.9		21.2	13.1	2.0
High	9.6	7.8	9.6	28.6	18.7		33.4	27.3	

Note: values shown in bold are statistically significantly different among those sociodemographic portions according to chi-square test for (*p* < 0.05).

A total of 52%–73% of surveyed consumers in all locations stored raw meat in the freezer (Table 6). Other consumers stored raw meat on the top or middle shelf of the refrigerator (16% of Indian, 17% of Korean, and 21% of surveyed Thai consumers), or elsewhere in the refrigerator. Similar findings were reported by Godwin and Coppings [29] for US consumers’ raw meat storage practices. The top and middle shelf and the door of the refrigerator are more likely to have elevated temperatures and storing temperature-sensitive foods is not recommended in these locations [25]. In addition these locations are more likely to contaminate foods on lower shelves of the refrigerator [35]. The potential for poultry juice dripping onto items on lower shelves is a problem that seemed to exist in all countries studied. Thus far, none of the studies have looked at the type of refrigerator shelving (meshed or glass, for example) to determine cross-contamination likelihood. Raw foods often are a source of bacterial pathogens. Vindigni *et al.* [26] conducted a study in Bangkok, Thailand, to determine bacterial pathogens on raw food samples. They found *Enterococcus* spp. on 94%, and *Salmonella* spp. on 61%, of the samples.

**Table 6:** Consumers’ raw meat, seafood, and poultry refrigeration and freezing practices. Where would you store the raw meat in the refrigerator? Available options included: Top shelf (1), middle shelf (2), bottom shelf (3), drawer (4), door (5), wherever there is room (6), on the counter or in the cabinet (7), in the freezer (8).

Demographic segmentation, %	India (*n* = 115)								Korea (*n* = 101)						Thailand (*n* = 100)						
	1	2	3	4	5	6	7	8	1	2	3	4	6	7	8	1	2	3	4	6	8
Total	7.0	9.6	1.7	2.6	0.9	2.6	3.5	72.2	9.9	7.9	7.0	8.9	11.9	2.0	52.4	17.0	4.0	2.0	3.0	1.0	73.0
*Gender*																					
Male									1.0		2.0	1.0	4.0		6.9	5.0	3.0			1.0	18.0
Female	7.0	9.6	1.7	2.6	0.9	2.6	3.5	72.2	8.9	7.9	5.0	7.9	7.9	2.0	45.5	12.0	1.0	2.0	3.0		55.0
*Age*																					
<35	**1.7**	**3.5**	**0.9**	**0.9**	**0.9**	**0.9**	**0.9**	**19.1**	2.0	5.9	4.0	2.0	5.0		15.8	2.0	1.0		1.0	1.0	8.0
>35	**5.2**	**6.1**	**0.9**	**1.7**		**1.7**	**2.6**	**53.0**	7.9	2.0	3.0	6.9	6.9	2.0	36.6	15.0	3.0	2.0	2.0		65.0
*Education*																					
Less than college	**2.6**	**3.5**		**0.9**			**2.6**	**12.2**	3.0	2.0	1.0	1.0	4.0	2.0	25.7		**2.0**	**1.0**		**1.0**	**19.0**
Some college courses or more	**4.3**	**6.1**	**1.7**	**1.7**	**0.9**	**2.6**	**0.9**	**60.0**	6.9	5.9	5.9	7.9	7.9		26.7	**17.0**	**2.0**	**1.0**	**3.0**		**54.0**
*Income*																					
Low	**6.1**	**5.2**	**0.9**	**1.7**	**0.9**	**0.9**	**3.5**	**27.0**	**3.0**	**1.0**		**1.0**	**2.0**	**2.0**	**14.9**						3.0
Medium	**0.9**	**3.5**	**0.9**			**0.9**		**20.9**	**4.0**	**1.0**	**3.0**	**2.0**	**7.9**		**14.9**	5.0		2.0		1.0	28.0
High		**0.9**		**0.9**		**0.9**		**24.3**	**3.0**	**5.9**	**4.0**	**5.9**	**2.0**		**22.8**	12.0	4.0		3.0		48.0

Notes: options 7 and 8 in Thailand and 5 in Korea were not checked by any consumers; values shown in bold are statistically significantly different among those sociodemographic portions according to chi-square test for (*p* < 0.05).

Although surveyed consumers stored raw meat on the refrigerator shelf, only 22%–30% placed something underneath the meat, probably to avoid spilling or dripping of meat juices (Table 7). This behavior trend was similar regardless of gender, age, education level, or income of the consumers in all the countries studied. These juice-catching utensils included plates (*n* = 22 in Korea, *n* = 32 in India, *n* = 6 in Thailand) and paper toweling or meshed bowls, with an additional plate or plastic bags (data not shown).

**Table 7:** Consumers’ raw meat, seafood, and poultry storage behavior. Would you place something under the raw meat in the refrigerator (Yes/No)?

Demographic segmentation, %	India (*n* = 115)		Korea (*n* = 101)		Thailand (*n* = 100)	
	Yes	No	Yes	No	Yes	No
Total	30.4	69.6	31.7	68.3	28.0	72.0
*Gender*						
Male			3.0	11.9	5.0	22.0
Female	30.4	69.6	28.7	56.4	23.0	50.0
*Age*						
<35	**7.0**	**21.7**	**7.9**	**26.7**	**4.0**	**9.0**
>35	**23.5**	**47.8**	**23.8**	**41.6**	**24.0**	**63.0**
*Education*						
Less than college	**6.1**	**15.7**	**18.8**	**19.8**	**10.0**	**13.0**
Some college courses or more	**24.3**	**53.9**	**12.9**	**48.5**	**18.0**	**59.0**
*Income*						
Low	**13.0**	**33.0**	7.9	15.8	**1.0**	**2.0**
Medium	**9.6**	**17.4**	10.9	21.8	**13.0**	**23.0**
High	**7.8**	**19.1**	12.9	30.7	**14.0**	**47.0**

Note: values shown in bold are statistically significantly different among those sociodemographic portions according to chi-square test for (*p* < 0.05).

### 3.2. Preparation Practices

The main contaminants while preparing food at home are hands, knives, and cutting boards [3]. This was supported by findings in our survey (Table 8). A total of 31% of participating Korean, 24% of Indian, and 30% of Thai consumers used the same cutting board for different foods, such as meats and vegetables and either did nothing or only wiped the cutting board between different foods. Indian consumers with higher than college education were significantly more likely (*p* = 0.01) to wash the cutting board in between cutting different foods or use a different cutting surface for different types of foods. Only a few consumers did not use a cutting surface when cutting foods (8% in India and 1% in Korea). Observational studies that would look at the washing process of the cutting board and the rate of success in removing raw meat particles from the cutting board or the order in which consumers cut up food ingredients using the same cutting board during cooking might be of interest.

**Table 8:** Consumers’ practices related to the use of cutting boards. When you are cutting various types of food such as meat, vegetables, eggs, bread, do you usually use: (Select one). Available options included: The same cutting surface (counter, plate, cutting board) and wipe or wash it at the end (1); The same cutting surface (counter, plate, cutting board) and wipe it between uses (2); The same cutting surface (counter, plate, cutting board) and wash it between uses (3); A different cutting surface for each type of food (4); Do not use a cutting surface (5).

Demographic segmentation, %	India (*n* = 115)					Korea (*n* = 97)					Thailand (*n* = 100) *			
	1	2	3	4	5	1	2	3	4	5	1	2	3	4
Total	12.2	12.2	44.3	22.6	8.7	19.6	12.3	36.1	31.0	1.0	12.0	18.0	43.0	27.0
*Gender*														
Male						4.1	1.0	5.2	5.2		7.0	4.0	11.0	5.0
Female	12.2	12.2	44.3	22.6	8.7	15.5	11.3	30.9	25.8	1.0	5.0	14.0	32.0	22.0
*Age*														
<35	1.7	3.5	16.5	4.3	2.6	6.2	3.1	12.4	14.4		2.0	4.0	6.0	1.0
>35	10.4	8.7	18.3	18.3	6.1	13.4	9.3	23.7	16.5	1.0	10.0	14.0	37.0	26.0
*Education*														
Less than college	**4.3**	**1.7**	**5.2**	**5.2**	**5.2**	9.3	6.2	12.4	9.3		3.0	7.0	8.0	5.0
Some college courses or more	**7.8**	**10.4**	**39.1**	**17.4**	**3.5**	10.3	6.2	23.7	21.6	1.0	9.0	11.0	35.0	22.0
*Income*														
Low	**7.8**	**6.1**	**13.9**	**11.3**	**7.0**	4.1	6.2	9.3	2.1			1.0		2.0
Medium	**2.6**	**5.2**	**12.2**	**5.2**	**1.7**	7.2	2.1	11.3	13.4		4.0	4.0	20.0	8.0
High	**1.7**	**0.9**	**18.3**	**6.1**		8.2	4.1	15.5	15.5	1.0	8.0	13.0	23.0	17.0

Notes: values shown in bold are statistically significantly different among those sociodemographic portions according to chi-square test for (*p* < 0.05). * None of the consumers in Thailand selected the option “Do not use a cutting surface”.

Washing hands with soap and water after handling raw meat or eggs seemed to be the favored approach for more than half of surveyed consumers in India and Thailand, and a third of consumers in Korea (Table 9). However Sudershan *et al.* [36] found that most Indian women would wash their hands with water, but not with soap. Premakumari *et al.* [37] found 81% of consumers in India would wash their hands before eating. However, access to clean water may be restricted. A total of 22% of consumers in India, 38% in Korea, and 32% in Thailand would wash their hands with water, but not use soap (Table 9). According to DeDonder *et al.* [38] this would be insufficient to avoid cross-contamination. In Thailand, female respondents were more likely to wash their hands using soap than were male respondents. Furthermore, what consumers reported in our study may not reflect their actual practices. In a survey of hand-washing, and a follow-up observational study in Korea, about 60% of female college students claimed to use soap while washing hands, but only 0.9% used soap during the observational phase [39].

**Table 9:** Consumer practice of handling raw meat, poultry, seafood, or eggs. What was the first thing you did immediately after you handled these raw foods? Available options included: I cut up some other foods (1); I got other foods ready for cooking, but did not cut them up (2); I picked up a pot or pan to cook food (3); I wiped my hands off with a paper towel, dish cloth, or on my apron or clothing (4); Continued cooking without wiping, rinsing, or washing hands (5); Rinsed off my hands, but did not use soap (6); Washed hands with soap and water (7); Do not prepare raw meat, poultry, seafood, or eggs (8).

Demographic segmentation, %	India (*n* = 115)					Korea (*n* = 101)							Thailand (*n* = 100)						
	1	2	4	6	7	1	2	3	4	5	6	7	1	2	3	4	6	7	8
Total	0.9	2.6	7.8	22.6	66.1	4.0	3.0	8.9	5.0	3.0	38.7	37.6	1.0	3.0	2.0	7.0	32.0	52.0	3.0
*Gender*																			
Male						1.0		2.0			4.0	7.9		**3.0**		**5.0**	**10.0**	**9.0**	
Female	0.9	2.6	7.8	22.6	66.1	3.0	3.0	6.9	5.0	3.0	34.7	29.7	**1.0**		**2.0**	**2.0**	**22.0**	**43.0**	**3.0**
*Age*																			
<35	0.9		3.5	4.3	20.0			4.0	1.0		14.9	14.9		1.0			5.0	7.0	
>35		2.6	4.3	18.3	46.1	4.0	3.0	5.0	4.0	3.0	23.8	22.8	1.0	2.0	2.0	7.0	27.0	45.0	3.0
*Education*																			
Less than college		1.7	2.6	3.5	13.9	2.0	2.0	4.0	3.0	3.0	13.9	10.9		1.0		1.0	7.0	13.0	1.0
Some college courses or more	0.9	0.9	5.2	19.1	52.2	2.0	1.0	5.0	2.0		24.8	26.7	1.0	2.0	2.0	6.0	25.0	39.0	2.0
*Income*																			
Low	0.9	2.6	5.2	10.4	27.0	2.0	1.0	2.0	2.0	2.0	7.9	6.9						3.0	
Medium			0.9	7.8	18.3	1.0	1.0	2.0	2.0	1.0	9.9	15.8	1.0	1.0		3.0	9.0	21.0	1.0
High			1.7	4.3	20.9	1.0	1.0	5.0	1.0		20.8	14.9		2.0	2.0	4.0	23.0	28.0	2.0

Notes: values shown in bold are statistically significantly different among those sociodemographic portions according to chi-square test for (*p* < 0.05); none of the consumers selected options 3, 5, or 8 in India, 8 in Korea, and 5 in Thailand.

A total of 75% of surveyed consumers in India, 46% in Korea, and 48% in Thailand reported washing raw poultry before cooking, while 16% of Indian and 11% of Korean consumers reported washing raw eggs (Table 10). In addition, 48% of Thai consumers reported always washing both poultry and eggs. According to the Food Safety and Inspection Service (FSIS) [40] washing raw poultry or meats and eggs is not recommended as it is likely to cross-contaminate kitchen surfaces and utensils, which may lead to food-borne illnesses. It is reasonable, however, for the consumer to expect keeping food safe by washing poultry and eggs, especially if food has been acquired from a market or directly from farmer and visibly is not clean. In addition, readily available information sources, such as cookbooks or cooking shows often recommend washing chicken and other meat.

**Table 10:** Raw poultry and eggs preparation practice. Do you wash raw poultry/eggs before cooking them? Available options included: Yes, I always wash raw poultry (1); Yes, I always wash eggs (2); Yes, I always wash poultry and eggs (3); Sometimes—if they seem dirty (4); No (5).

Demographic segmentation, %	India (*n* = 133) *				Korea (*n* = 101) **				Thailand (*n* = 100)				
	1	2	4	5	1	2	4	5	1	2	3	4	5
Total	75.2	16.5	5.3	3.0	46.6	11.9	24.8	16.9	48.0	3.0	45.0	3.0	1.0
*Gender*													
Male					5.0	3.0	4.0	3.0	15.0		9.0	2.0	1.0
Female	75.2	16.5	5.3	3.0	41.6	8.9	20.8	13.9	33.0	3.0	36.0	1.0	
*Age*													
<35	22.6	1.5	1.5	0.8	15.8	1.0	12.9	5.0	7.0		5.0		1.0
>35	52.6	15.0	3.8	2.3	30.7	10.9	11.9	11.9	41.0	3.0	40.0	3.0	
*Education*													
Less than college	**22.5**	**3.0**		**1.5**	14.9	5.0	9.9	8.9	10.0	1.0	12.0		
Some college courses or more	**57.9**	**13.5**	**5.3**	**1.5**	31.7	6.9	14.9	7.9	38.0	2.0	33.0	3.0	1.0
*Income*													
Low	33.8	3.0	2.3	2.3	8.9	3.0	6.9	5.0	3.0				
Medium	20.3	6.8	0.8	0.8	14.9	4.0	9.9	4.0	11.0	1.0	24.0		
High	21.1	6.8	2.3		22.8	5.0	7.9	7.9	34.0	2.0	21.0	3.0	1.0

Notes: values shown in bold are statistically significantly different among those sociodemographic portions according to chi-square test for (*p* < 0.05). * Option 3 in India was “I do not cook raw poultry or eggs” and none of the consumers in India selected option 3. ** None of the consumers in Korea selected the option “Yes, I always wash raw poultry and eggs”.

Temperature plays an important part in storing home-made mayonnaise and home-made salads with mayonnaise [41]. Those authors found that *Salmonella enteritidis* was able to grow rapidly in salads and mayonnaise that were stored at 25 °C, but not those stored at 10 °C. *Listeria monocytogenes*, however is able to grow in contaminated pasta and egg salads, even in cold storage [42]. FSIS [43] recommends cold perishable foods be refrigerated within 2 h from preparation. According to this study the majority of surveyed consumers would practice this behavior in India, Korea, and Thailand (Table 11). However, a large portion (38%) of Indian consumers reported leaving leftovers from a freshly prepared salad that contains mayonnaise or eggs unrefrigerated for more than 4 h. This behavior likely is associated with local food culture and habits. This is an area where local food authorities need to further educate consumers.

**Table 11:** Leftover handling practice. The last time you had leftovers from a freshly prepared salad that contained eggs or mayonnaise, how long did you let the leftovers sit at room temperature before you put them in the refrigerator or ate them later without refrigeration? Available options included: 1 hour or less (1); More than 1 hour, but less than 2 hours (2); More than 2 hours, but less than 3 hours (3); More than 3 hours, but less than 4 hours (4); 4 hours or more (5).

Demographic segmentation, %	India (*n* = 115)					Korea (*n* = 100) *				Thailand (*n* = 100)				
	1	2	3	4	5	1	2	3	5	1	2	3	4	5
Total	34.8	13.9	7.0	6.1	38.3	71.0	16.0	8.0	6.0	74.0	12.0	7.0	4.0	3.0
*Gender*														
Male						11.0	4.0			**16.0**	**8.0**	**2.0**	**1.0**	
Female	34.8	13.9	7.0	6.1	38.3	60.0	12.0	8.0	6.0	**58.0**	**4.0**	**5.0**	**3.0**	**3.0**
*Age*														
<35	11.3	4.3	0.9	4.3	7.8	24.0	4.0	2.0	5.0	**5.0**	**6.0**	**2.0**		
>35	23.5	9.6	6.1	1.7	30.4	47.0	12.0	6.0	1.0	**69.0**	**6.0**	**5.0**	**4.0**	**3.0**
*Education*														
Less than college	3.5	2.6	2.6	2.6	10.4	**22.0**	**8.0**	**7.0**	**2.0**	17.0	4.0	2.0		
Some college courses or more	31.3	11.3	4.3	3.5	27.8	**49.0**	**8.0**	**1.0**	**4.0**	57.0	8.0	5.0	4.0	3.0
*Income*														
Low	18.3	7.0	4.3	5.2	11.3	**11.0**	**6.0**	**5.0**	**2.0**	3.0				
Medium	8.7	4.3	0.9		13.0	**27.0**	**2.0**	**1.0**	**3.0**	26.0	6.0	2.0	1.0	1.0
High	7.8	2.6	1.7	0.9	13.9	**33.0**	**8.0**	**2.0**	**1.0**	45.0	6.0	5.0	3.0	2.0

Notes: values shown in bold are statistically significantly different among those sociodemographic portions according to chi-square test for (*p* < 0.05). * No consumers in Korea selected option 4.

Sudershan *et al.* [44] pointed out the limited number of studies that address consumer food safety related behavior characteristics in India. Furthermore, studies have found low levels of food safety knowledge among consumers. Sudershan *et al.* [36] looked at women with children younger than five years old in India. These authors found that food safety related practices are mostly taught from mother to daughter, and are considered important. However, there were behaviors identified that may cause foodborne illnesses. For example, in India, all eggs are purchased at room temperature because small and large grocery stores keep eggs at room temperature due to a lack of sufficient cold storage facilities. It has been reported, in India, that over 99% of food and grocery is sold by traditional retailers (Kirana stores, street hawkers, and wet market stall operators) and only 5% of all poultry output is marketed in processed form [45]. In the Indian and Thai context, refrigeration is all the more necessitated by tropical climatic conditions, which are conducive to faster microbial growth. A study on food safety practices in India revealed that over 80% of the consumers stored cooked foods at room temperature because only 19% owned refrigerators. Usage of refrigerators for storing leftover cooked non-vegetarian food was important among literate respondents and those with high standards of living [46]. 

According to Henley *et al.* [9] consumers with different cultural backgrounds may have specific approaches to food handling. These authors found that even though there are some overall hazardous behaviors, such as not using a thermometer and thawing foods at room temperature, there were culture-specific characteristics as well. An example of this behavior would include cutting poultry into smaller, bite-sized pieces in some cultures that could lead to cross-contamination issues. Our results confirmed the potentially contaminating behavior, such as washing raw eggs and poultry, and using the same cutting surface for multiple foods without washing in between.

The limitations of this study include a relatively small geographic representation of surveyed consumers in each country. The approximately 100 consumers in each country came from only one or two cities, which is common in many surveys. Some previous studies, in these and other regions have included a smaller number of respondents or participants [38,46]. Nevertheless, these results should not be considered as representative of the whole countries of India, Korea, or Thailand. One potential perceived limitation is the low number of men surveyed. However, this was caused by the cultural background of Asian countries, where male and female roles are somewhat rigidly defined, and men may not purchase, prepare, or store foods. Low, or nonexistent, male participant rates have been observed in other studies conducted with minorities [9] or in Asian countries [17,46].

Although limitations are present, the data serves as a first look at what likely are problem areas for food safety handling of poultry and eggs—lack of refrigerated storage for eggs, cross contamination issues during preparation, and longer than recommended times for leftovers held at room temperature in some populations. The study also suggests that food safety education may be needed, especially with younger populations that have less exposure to preparation and storage and are still developing life-long food safety habits. In addition, the collection of data in a uniform manner across three Asian countries enables immediate comparison of results across countries.

## 4. Conclusions

This study compared consumers’ purchase, storage, handling, and preparation of poultry products and eggs in three Asian countries: India, Korea, and Thailand. The results indicated similar patterns in purchase and storage behaviors among consumers in all three countries. For example most consumers would store raw eggs in the refrigerator, but some stored cooked eggs at room temperature. In addition most consumers froze raw meat, poultry, or seafood. However, some consumers who stored raw meat in the refrigerator would do this on the top or middle shelf and this could potentially lead to meat juice contaminating other foods. Most consumers claimed to wash hands with soap and water; however there were portions of consumers who did not. Consumers reported practices that support cross-contamination during cooking, such as washing raw poultry and eggs, inadequately washing cutting surfaces between foods, and not refrigerating dishes that could become a food hazard. Health education in food safety must be designed to suit prevailing socioeconomic conditions and specific cultural groups in different countries. The findings from this study indicate a need for raising consumer awareness about food safety issues. The similarity of these findings across three Asian countries suggests food safety educational efforts may benefit from collaborative efforts among universities, health educators, and food safety authorities. 
